# Learn, adapt, act: A pragmatic approach for intervening on disparities in hepatocellular carcinoma outcomes

**DOI:** 10.1097/HC9.0000000000000665

**Published:** 2025-02-26

**Authors:** Tiana Walker, Lauren D. Nephew

**Affiliations:** 1 Department of Medicine, Massachusetts General Hospital, Boston, Massachusetts, USA; 2 Department of Medicine, Division of Gastroenterology and Hepatology, Indiana University, Indianapolis, Indiana, USA

Social and structural determinants of health (SSDoH) are well established as critical factors influencing well-being and health care outcomes.^1^,^2^ SSDoH encompasses multiple levels of influences, including health care policies (eg, insurance affordability and coverage), structural elements (eg, neighborhood social cohesion, food security, accessible transportation, and educational attainment), provider-level factors (eg, implicit bias), and patient-specific factors (eg, medical mistrust and health literacy).^1^,^2^ These interconnected determinants create a tiered health care system that results in differential outcomes, often based on race, ethnicity, and socioeconomic status.

In the United States, racial and ethnic health disparities are evident across many diseases, including HCC, where risk factors such as cirrhosis are deeply intertwined with lower socioeconomic status. Black individuals in the United States are disproportionately affected by social deprivation, contributing to pronounced disparities in HCC screening, early detection, treatment, and mortality.^3^ Identifying the specific SSDoH that mediates health outcomes and access in HCC is essential to designing targeted interventions that improve equity and reduce disparities.

Two recent studies provide additional insights into the role of SSDoH in HCC care and outcomes.^4^,^5^ In a multicenter analysis, Rich and colleagues found that Black and Hispanic patients were more likely to present at later HCC stages compared to non-Hispanic White and Asian patients. Adjusting for individual demographic and clinical factors, health care engagement, insurance, and area-level SSDoH mitigated stage disparities for Hispanic patients but not for Black patients. Disparities in survival between Black and White patients also persisted after adjusting for individual clinical and demographic factors. However, these disparities attenuated with the addition of HCC stage and treatment, suggesting that these factors largely drive survival disparities.^4^

Singal and colleagues explored the relationship between SSDoH, receipt of HCC surveillance, curative treatment, and cost using Medicare Fee-for-Service data alongside claims from commercial and managed Medicaid sources. White race, younger age, and favorable area-level SSDoH were associated with greater uptake of surveillance, while Black patients were less likely to receive ultrasound surveillance. Patients who underwent surveillance were more likely to receive curative treatment, illustrating a critical link between surveillance and treatment outcomes. Higher health care costs were independently associated with Black race, low English proficiency, living alone, and inversely associated with CT/MRI-based surveillance. This underscores the economic burden of unchecked health disparities in HCC care.^5^

Rich and Singal emphasize the significant role of SSDoH across the HCC care continuum, from surveillance to treatment. They advocate for structural and large-scale policy changes to mitigate disparities, including expanding health insurance coverage, increasing the capacity of primary and specialty care providers in underserved areas, addressing economic barriers, such as transportation and opportunity costs associated with surveillance and treatment, implementing neighborhood-level interventions to address food insecurity, lack of green spaces, and safety concerns.

These policy-driven solutions are necessary to eliminate health disparities across diseases. But what role does hepatology specifically play in HCC health care disparity elimination? Policy-driven, systemic solutions are essential but insufficient on their own to eliminate health care disparities. Achieving equity requires multilevel interventions that address policy and health system, clinic, provider, and patient-level determinants of health.

We must do our part and have enough knowledge to act now. The disparities in HCC care and outcomes are well documented, and it is evident that minoritized and marginalized populations are not receiving evidence-based care.^3^ Health system, clinic, and provider-based approaches to improve disparities in HCC outcomes could include embedding refining patient navigation programs, providing implicit bias training to office, clinic staff, and providers, delivering equity-centered performance feedback, optimizing clinic workflows for high-risk populations, leveraging culturally tailored educational materials, and exploring the sustainability of flexible appointment options (Table 1). In addition, we must leverage our understanding of individual and area-level determinants of health in HCC to target these often expensive and labor-intensive interventions to the populations at greatest risk (Table 1). Many of these approaches have already been explored in other medical domains—such as breast cancer, lung cancer, and hypertension.^6^,^7^,^8^ They can be methodologically adapted to meet the unique needs and contexts of patients with HCC, accelerating progress toward health equity.

**TABLE 1:** Suggested interventions to improve racial, ethnic, and socioeconomic disparities in HCC outcomes

Level within healthcare﻿	Intervention﻿	Details﻿
Health system﻿	Risk-stratification and patient identification	Implement evidence-based, system-wide protocols to identify patients with social risk factors (ie, census-tract) to target for intervention.
Health system	Incentives	Provide provider incentives for closing racial and SDOH gaps in screening; improving health disparities related health care cost.
Clinic	Patient Navigation Programs	Employ patient navigators for patients from high-risk cohorts, to assist with scheduling, follow-ups, and addressing logistical barriers.
Clinic	Clinic Workflow Optimization	Develop streamlined processes to schedule screening at the same time as routine visits.
Clinic	Culturally Tailored Education	Provide educational materials tailored to cultural and linguistic needs in multimedia formats.
Clinic	Flexible Appointment Options	Offer extended hours and weekend appointments for screening and appointments.
Provider	Implicit Bias Training	Implement training to reduce implicit biases and encourage consistent application of protocols.
Provider	Performance Feedback	Provide regular feedback on screening rates stratified by SSDoH and incentivize improvements.
Provider	Shared Decision-Making Tools	Develop tools to facilitate patient conversations and address concerns in a culturally sensitive manner.

Abbreviation: SSDoH, social and structural determinants of health.

Adaptation, a core concept in implementation science, is the thoughtful and deliberate process of altering an intervention’s design or delivery to enhance its fit or effectiveness in a specific context.^9^ While adaptation offers the potential to improve outcomes by aligning interventions with the needs of a target population or system, it is not without challenges. Successful adaptation demands careful balance; when done well, it can increase effectiveness and promote equity. However, modifications that remove essential elements of an intervention or fail to address the population’s unique needs may reduce effectiveness and inadvertently worsen health disparities.^9^

The FRAME^9^ (Framework for Reporting Adaptations and Modifications to Evidence-based Interventions) framework provides a structured method for adapting evidence-based interventions (Figure 1). It prompts us to learn from proven approaches and apply user-centered design to adapt targeted solutions. The FRAME steps include:

1. Learn from evidence: Document core components of successful interventions to preserve fidelity.
2. Adapt for HCC: User-centered design to modify intervention content and delivery to meet disease-specific needs and local contexts.
3. Implement and test: Apply adapted models and accelerate this implementation through hybrid type II trials, enabling the simultaneous evaluation of intervention effectiveness, impact on health disparities, and implementation across diverse settings.^10^
4. Refine based on feedback: Evaluate outcomes and use findings to refine interventions further.

**FIGURE 1:**
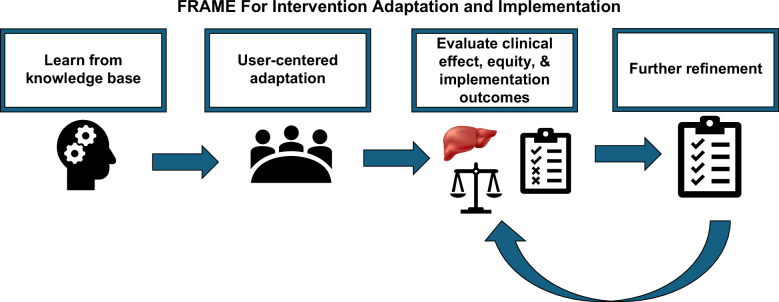
FRAME for intervention adaptation and implementation. Abbreviation: FRAME, Framework for Reporting Adaptations and Modifications to Evidence-based Interventions.

## CONCLUSIONS

While advocating for systemic policy changes, we must also leverage, adapt, and implement evidence-based interventions and refine them for sustained impact within our health systems and practices. If we aim to eliminate health disparities in hepatology, no area is better suited as a starting place than HCC, given the wealth of emerging knowledge about SSDoH and adaptable lessons available. By taking these steps, health care systems, providers, and researchers can drive meaningful progress in HCC-related health equity.
